# Disease Associated Mutations in K_IR_ Proteins Linked to Aberrant Inward Rectifier Channel Trafficking

**DOI:** 10.3390/biom9110650

**Published:** 2019-10-25

**Authors:** Eva-Maria Zangerl-Plessl, Muge Qile, Meye Bloothooft, Anna Stary-Weinzinger, Marcel A. G. van der Heyden

**Affiliations:** 1 Department of Pharmacology and Toxicology, University of Vienna, 1090 Vienna, Austria; eva-maria.zangerl@univie.ac.at (E.-M.Z.-P.); anna.stary@univie.ac.at (A.S.-W.); 2 Department of Medical Physiology, Division of Heart & Lungs, University Medical Center Utrecht, 3584 CM Utrecht, The Netherlands; M.Qile@umcutrecht.nl (M.Q.); meye10@hotmail.com (M.B.)

**Keywords:** inward rectifier channel, trafficking, alignment, mutation, *KCNJ*, K_IR_, disease, structure

## Abstract

The ubiquitously expressed family of inward rectifier potassium (K_IR_) channels, encoded by *KCNJ* genes, is primarily involved in cell excitability and potassium homeostasis. Channel mutations associate with a variety of severe human diseases and syndromes, affecting many organ systems including the central and peripheral neural system, heart, kidney, pancreas, and skeletal muscle. A number of mutations associate with altered ion channel expression at the plasma membrane, which might result from defective channel trafficking. Trafficking involves cellular processes that transport ion channels to and from their place of function. By alignment of all K_IR_ channels, and depicting the trafficking associated mutations, three mutational hotspots were identified. One localized in the transmembrane-domain 1 and immediately adjacent sequences, one was found in the G-loop and Golgi-export domain, and the third one was detected at the immunoglobulin-like domain. Surprisingly, only few mutations were observed in experimentally determined Endoplasmic Reticulum (ER)exit-, export-, or ER-retention motifs. Structural mapping of the trafficking defect causing mutations provided a 3D framework, which indicates that trafficking deficient mutations form clusters. These “mutation clusters” affect trafficking by different mechanisms, including protein stability.

## 1. Introduction 

Seventy years ago, Katz detected the inward rectification phenomenon for the first time [1]. Its unexpected property of conducting larger inward than outward potassium currents at similar deviations from the potassium equilibrium potential was unprecedented at that time. During the following decades, the understanding of inward rectifier channels was established further, stimulated by biophysical analysis and cloning of K_IR_ genes. Inward rectifying channels—unlike voltage-gated potassium channels (K_v_) which open in response to alterations in transmembrane electrostatic potential [2,3]—are primarily gated by intracellular substances (e.g., polyamines and Mg^2+^). Spermine and spermidine—two polyamines for which micromolar concentrations are sufficient to reach physiological effective levels—cause stronger block of the outward current than Mg^2+^. The underlying molecular mechanism of rectification was first explained by Lopatin in 1994 [3]. Polyamines enter the channel pore from the cytoplasmic side and subsequently interact with six specific residues (i.e., K_IR_2.1 E224, D259, E299, F254, D255 and D172) [4] in the transmembrane pore domain and its cytosolic pore extension. A similar mechanism of pore-blocking is caused by Mg^2+^, but weaker.

The inward rectifier channel family consists of strong and weak rectifiers. Strong rectifiers, e.g., K_IR_2 and K_IR_3, are often expressed in excitable cells such as neuronal or muscle cells. Their rectifying properties enable cells to conserve K^+^ during action potential formation and facilitate K^+^ entry upon cell hyperpolarization. In addition, they contribute to repolarization and stabilization of the resting membrane potential. For example, application of 10 µM barium, at that concentration rather specific for K_IR_2 channel inhibition, lengthened the action potential of guinea-pig papillary muscle preparations by 20 ms [5]. In the heart, K_IR_2 is strongly expressed in the ventricles and less in the atrioventricular node (AVN) [6]; K_IR_3 is mainly expressed in the atrium with much lower levels in the ventricle. Weak rectifier channels, e.g., K_IR_1, K_IR_4, and K_IR_5, are mainly associated with potassium homeostasis and often regulate extracellular potassium concentrations to allow functioning of several ion (co)transporters.

K_IR_ channels are encoded by *KCNJ* genes. Various diseases associate with mutations in *KCNJ* genes. The aim of this review is to correlate disease associated mutations causing aberrant inward rectifier channel trafficking with protein domains important for trafficking by means of channel alignment, and finally to put mutational changes in a structural framework.

## 2. Classification, Structure, and Expression

**Figure 1:**
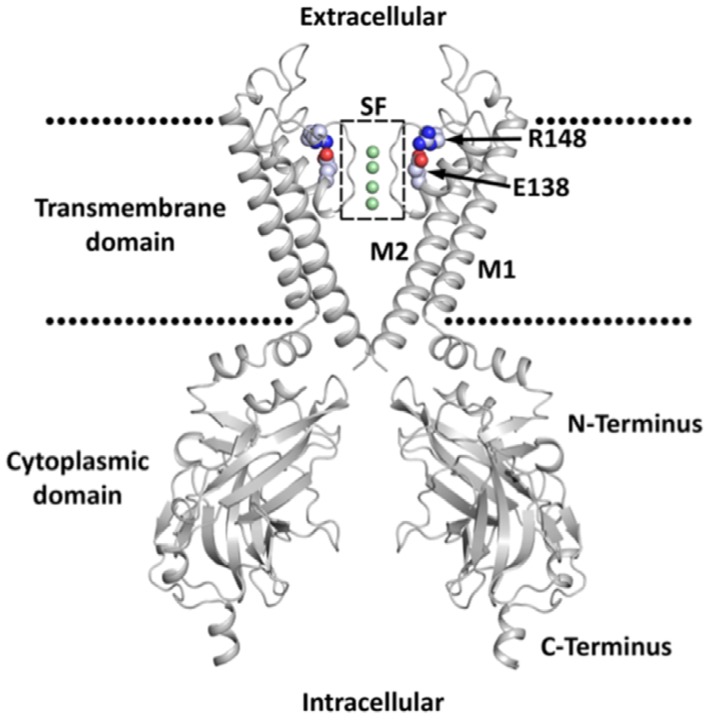
Two opposing domains of the K_IR_ channel with structural common features highlighted. The membrane is indicated by dotted lines. The selectivity filter (SF) is highlighted by a dotted box. Ions inside the SF are shown as green spheres. Residues E138 and R148 (K_IR_2.1) are shown as spheres.

The K_IR_ family is divided into seven subfamilies (K_IR_1-7) according to their amino-acid homology [4]. The sequence homology is 40% between subfamilies and rises to 70% within some subfamilies. The structural common features of these channels are that the channel pore is formed by a tetramer of subunits, most often homotetramers (Figure 1). Each subunit has two transmembrane domains (M1 and M2) which are separated by a pore-loop that contains the GYG (or GFG) potassium selectivity filter motif located close to the extracellular side of the membrane. Pore-loop stability depends strongly on one negatively and one positively charged residue, E138 and R148 respectively in K_IR_2.1 [7]. There is a relatively short N-terminus linked to M1 and a longer C-terminus linked to M2 which form the characteristic cytoplasmic extended pore domain (CTD). Despite their structural similarities, the K_IR_ subfamilies also display divergent properties, e.g., sensitivity to extracellular Ba^2+^ or the response to regulatory signals.

All K_IR_ family members have a widespread expression pattern [4]. Neural tissues strongly express K_IR_2, K_IR_3, K_IR_4, and K_IR_5. Kidneys show high expression of K_IR_4, K_IR_5, and K_IR_6, whereas pancreatic tissue highly expresses K_IR_5 and K_IR_6. The retina shows profound expression of K_IR_7. The heart displays strong expression of K_IR_2, K_IR_3 and K_IR_6. K_IR_2 subfamily-members form the classical I_K1_ current in working ventricular and atrial cardiomyocytes, where they contribute to repolarization and resting membrane potential stability. K_IR_3 (I_KAch_) members are strongly expressed in the nodal tissues of the heart, where they are involved in heart rate regulation [8]. Further, they are widely expressed in the brain where they have numerous neurological functions [9]. K_IR_6 channels form octamers with the ATP/ADP sensing SUR subunits and couple cellular metabolic status to cardiac repolarization strength.

## 3. Channel Trafficking

**Figure 2:**
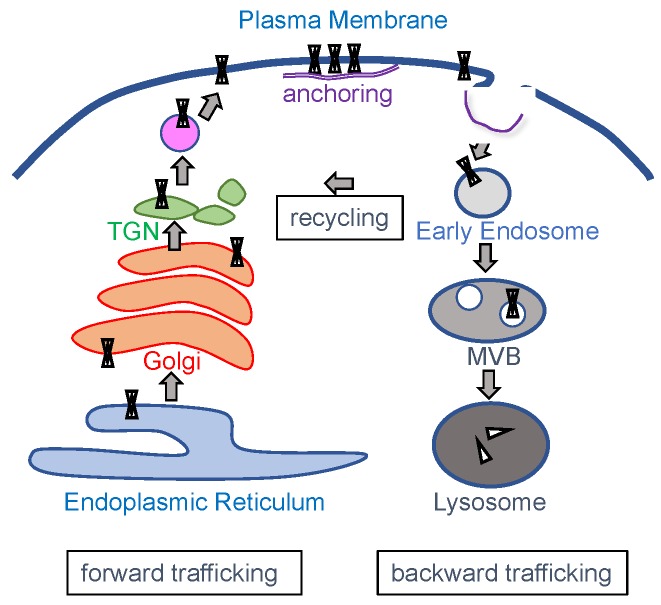
Schematic representation of intracellular trafficking pathways of K_IR_ channels. TGN, trans-Golgi network; MVB, multivesicular body.

Following their translation in the endoplasmic reticulum (ER), correctly folded K_IR_ channels are transported to the plasma membrane, a process known as forward trafficking, where they exert their main biological role (Figure 2). Upon removal from the plasma membrane, channel proteins can enter the degradation pathway in a process named backward trafficking. In addition, K_IR_ channels can enter several recycling pathways. Each of these processes is well regulated and depends mainly on specific trafficking motifs in the channels primary sequence in concert with specific interacting proteins that direct and/or support subsequent trafficking steps. Incorrectly folded proteins will enter the endoplasmic-reticulum-associated protein degradation pathway.

ER-export signals have been determined in several K_IR_ channels [10,11,12,13], see also Section 5.3, with homology between subfamily members (e.g., FCYENE in K_IR_2.x channels), although not always among other K_IR_ family members. Not all K_IR_ members possess an ER-export signal, and some might even restrict forward trafficking or stimulate lysosomal breakdown when part of a heteromeric channel, as seen for K_IR_3.3 [12]. Other channels even have ER-retention signals that only become masked upon proper channel assembly, as seen for the K_IR_6 family [13].

Trans-Golgi transport of several K_IR_ channels has been demonstrated to depend on interaction with Golgin tethers that reside in the trans-Golgi network. For example, Golgin-160 interacts with the C-terminal domain of K_IR_1.1 channels which results in increased forward trafficking and an increase in K_IR_1.1 currents [14]. In a similar fashion, Golgin-97 was shown to interact with the C-terminus of K_IR_2.1 and promotes transport to the Golgi-export sites [15]. Golgi-export signals have been characterized in a few K_IR_ channels [16,17]. By a combination of cytoplasmic N- and C-terminal domains, a so-called Golgi-export signal patch is formed that interacts with the AP-1 clathrin adaptor protein.

Protein motifs involved in backward trafficking have been studied less. K_IR_1.1 internalization depends on clathrin-dynamin mediated endocytosis which involved N375 in the K_IR_1.1 putative internalization motif NPN [18]. Internalized K_IR_1.1 channels depend on CORVET and ESCRT protein complexes for subsequent trafficking to the early endosome and the multivesicular body that eventually fuses with the lysosome, respectively [19]. K_IR_2.1 channels are regulated by the ESCRT machinery also [20]. It was demonstrated, by a pharmacological approach, that K_IR_2.1 degradation also depends on clathrin mediated endocytosis and lysosomal activity, and their inhibition resulted in enhanced I_K1_ currents [21,22]. The TPVT motif of the K_IR_5.1 channel protein binds the Nedd4-2 E3 ubiquitin ligase. In K_IR_5.1/K_IR_4.1 heteromeric complexes, this was suggested to result in ubiquitination and subsequent degradation of K_IR_4.1 in the proteasome [23].

Finally, trafficking, anchoring and plasma membrane localization of K_IR_ channels is regulated by their interaction with scaffolding proteins. The C-terminal K_IR_2.2 SEI PDZ-binding domain interacts with SAP97, PSD-95, Chapsyn-110, SAP102, CASK, Dlg2, Dlg3, Pals2, Veli1, Veli3, Mint1, and abLIM from rat brain lysates, and SAP97, CASK, Veli-3, and Mint1 from rat heart lysates [24,25,26]. Additionally, interactions between syntrophins, dystrobrevins and the K_IR_2.2 PDZ domain were shown by these authors. K_IR_2.1 and K_IR_2.3 also interact with SAP97 in the heart. Using an NMR approach, it was found that additional residues close to the K_IR_2.1 PDZ domain were involved in PSD-95 interaction [27]. Furthermore, PSD-95 interacts with K_IR_4.1 and K_IR_5.1 in the optic nerve and brain, and PSD-95 interaction is essential for K_IR_5.1 expression at the plasma membrane of HEK293 cells [28,29]. The C-terminal PDZ-binding motif SNV interacts with PSD-95, and K_IR_4.1 mediated current density more than doubled upon PSD-95 cotransfection in HEK293 cells, and increased even threefold upon SAP97 cotransfection [30]. Upon silencing of SAP97, the I_K1_ current decreased due to reduced plasma membrane expression of K_IR_2.1 and K_IR_2.2 ion channels [31]. Residues 307–326 of K_IR_2.1 are involved in interactions with the actin binding protein filamin A. Interestingly, these interactions are unaffected by the Andersen–Tawil deletion Δ314/315 [32]. In arterial smooth muscle cells, filamin A and K_IR_2.1 colocalize in specific regions of the plasma membrane. Although filamin A is not essential for K_IR_2.1 trafficking to the plasma membrane, its absence reduces the amount of K_IR_2.1 channels present at the plasma membrane [32].

Whereas this research field provided many new insights during the last two decades, one has to emphasize that no complete trafficking pathway for any K_IR_ channel protein has been deciphered in detail yet. Furthermore, most of our current knowledge is derived from ectopic expressions systems rather than human native tissue or cells. Currently, we cannot exclude that K_IR_ subtype and/or tissue specific pathways exist. The observations that several diseases associate with K_IR_ channel trafficking malfunction might help us to further understand K_IR_ protein trafficking processes in their natural environments in vivo.

## 4. Diseases and Syndromes Associated with K_IR_ Channel Dysfunction

A number of human diseases associate with mutations in K_IR_ channels, as indicated in Table 1. Bartter syndrome type II is a salt-losing nephropathy resulting in hypokalemia and alkalosis associated with loss-of-function mutations in K_IR_1.1 channel proteins. K_IR_1.1 channels are essential for luminal extrusion of K^+^ in the thick ascending limb of Henle’s loop, thereby permitting continued activity of the NKCC2 cotransporter important for sodium resorption [33]. Loss-of-function in K_IR_2.1 causes Andersen–Tawil syndrome characterized by periodic skeletal muscle paralysis, developmental skeletal abnormalities, as well as biventricular tachycardia with or without the presence of long QT. On the other hand, K_IR_2.1 gain-of-function mutations result in cardiac phenotypes, atrial fibrillation and short QT syndrome, explained by increased repolarization capacity and thus shortened cardiac action potentials [34,35]. Thyrotoxic hypokalemic periodic paralysis associated with K_IR_2.6 loss-of-function mutations affect skeletal muscle excitability under thyrotoxic conditions [36]. Keppen–Lubinsky syndrome is an extremely rare condition associated with K_IR_3.2 gain-of-function mutations. Its phenotype encompasses lipodystrophy, hypertonia, hyperreflexia, developmental delay and intellectual disability [37,38]. Familial hyperaldosteronism type III is associated with loss-of-function mutations in K_IR_3.4 channel proteins. The disease is characterized by early onset of severe hypertension and hypokalemia. Mutant K_IR_3.4 channels lack potassium specificity and the resulting inflow of Na^+^ and accompanying cell depolarization of zona glomerulosa cells increases intracellular Ca^2+^ concentrations, which activates transcription pathways that raise aldosterone production [39]. Loss-of-function mutations in K_IR_3.4 associate with long QT syndrome 13, which indicates that these acetylcholine activated channels, mostly known from nodal tissues, also play a role in ventricular repolarization processes [40]. EAST (epilepsy, ataxia, sensorineural deafness, tubulopathy)/SeSAME syndrome is a salt-losing nephropathy combined with severe neurological disorders. The disease associated loss-of-function mutations in K_IR_4.1 channels expressed in the distal convoluted tubule, result in hypokalemic metabolic acidosis. Impaired K_IR_4.1 function in glial cells will increase neural tissue potassium levels giving rise to neuron depolarization, whereas reduced potassium concentration in the endolymph affect cochlear hair cell function [41]. Cantú syndrome results from gain-of-function of I_KATP_ channels, either due to mutation in K_IR_6.1 or the SUR2 subunits. Many of these mutations decrease the sensitivity of the channel to ATP-dependent closure [42]. Insulin release by pancreatic beta-cells is regulated by their membrane potential and L-type Calcium channel activity. Depolarization activates Ca^2+^ influx inducing insulin release from intracellular stores into the extracellular fluid. Loss-of-function mutations in K_IR_6.2 result in membrane depolarization and thus insulin release and associate with hyperinsulism and hypoglycemia. Gain-of-function mutations on the other hand impair insulin release and associate with different forms of diabetes [43]. K_IR_7.1 channels are expressed in the apical membrane of retinal pigmented epithelial cells and contribute to K^+^ homeostasis in the subretinal space. Loss-of-function mutations in K_IR_7.1 associate with retinal dysfunction observed in Lever congenital amaurosis type 16 and Snowflake vitreoretinal degeneration [44].

In many of the above-mentioned diseases, loss-of-function has been associated with aberrant trafficking, most likely forward trafficking. Nonetheless, enhanced backward trafficking or impaired plasma-membrane anchoring cannot be excluded. However, most gain-of-function mutations are likely not related to trafficking abnormalities. Loss-of-specificity mutations, as seen in some K_IR_3.4 mutations, neither result from trafficking issues.

## 5. K_IR_ Protein Alignment of Trafficking Associated Disease Mutations

**Figure 3:**
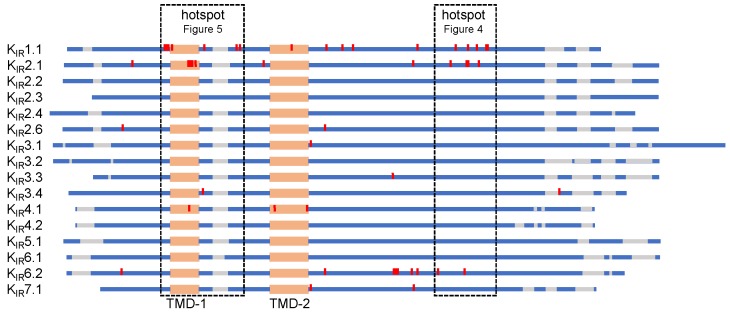
Schematic representation of inward rectifier channels (K_IR_1–7) sequence alignment. Red: mutations associated with aberrant trafficking; mutation hotspots are boxed. Orange: transmembrane domain; blue: K_IR_ protein sequence; gray: sequence gap.

In order to identify potential protein domains associated with K_IR_ trafficking defects in human disease, we aligned all K_IR_ isoforms and highlighted residues (in red) of which the mutations are experimentally proven to associate with trafficking defects (Figure 3, Supplementary Figure S1). Furthermore, additional mutations in other K_IR_ isoforms at homologues positions, but currently not related to impaired trafficking, are indicated (Supplementary Figure S1, in green). Trafficking associated mutations are found dispersed along the protein sequence, with one “hotspot” in the G-loop and adjacent C-terminal region, and one “hotspot” in and around transmembrane domain 1. Additionally, from a structural point of view, there is another “hotspot” at the immunoglobulin-like domain (IgLD), which is described in Section 6.1.

### 5.1. C-Terminal Trafficking Mutation Hotspot

**Figure 4:**
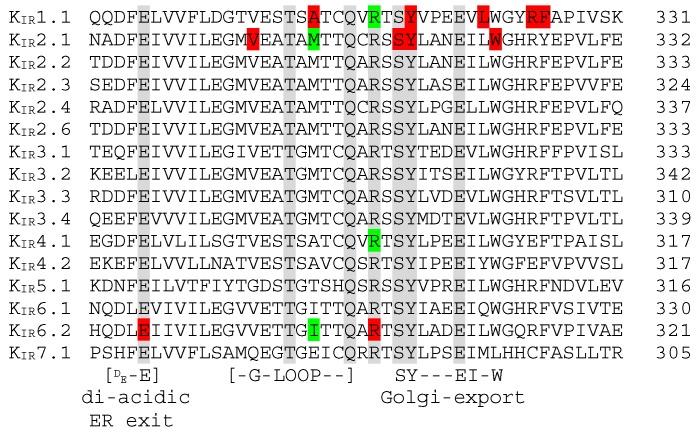
K_IR_1–7 sequence alignment of the C-terminal mutation hotspot. Red: Mutations associated with aberrant trafficking; Green: Residues whose mutations are currently not related to impaired trafficking. Numbers at the right refer to the last amino-acid residue in the respective sequence shown. Conserved residues among all K_IR_ members are shaded gray. Locations of the di-acidic ER exit, G-loop and the Golgi-Export signal sequence (see text) are indicated below the alignment.

Figure 4 depicts the alignment of the C-terminal hotspot, having ten trafficking associated mutations/deletions located in K_IR_1.1, K_IR_2.1 and/or K_IR_6.2 over a stretch of 44 residues. This region also covers the filamin A interaction domain of K_IR_2.1 (307–326) [32]. The loss-of-function E282K mutation in K_IR_6.2 is associated with congenital hyperinsulinism [45]. This mutation affects normal function of a highly conserved di-acidic ER exit signal (DxE) that prevents mutant channels to enter ER exit sites, which thus fail to traffic to the plasma membrane [45]. The Andersen–Tawil loss-of-function K_IR_2.1 mutation V302M is located in the G-loop and displays intracellular, but no plasma-membrane expression, upon transfection of HEK293 cells [46]. However, Ma et al., [47] demonstrated that the K_IR_2.1 V302M mutation does not affect trafficking. The Bartter syndrome associated loss-of-function A306T mutation in K_IR_1.1 is also located in the G-loop. Its expression in the *Xenopus* oocyte membrane is strongly reduced compared to wildtype channels [48]. At homologues positions, disease causing mutations have been found in K_IR_2.1 [49] and K_IR_6.2 [50] whose cause for loss- and gain-of-function, respectively, is unknown but may be caused by trafficking abnormalities. Hyperinsulinism associated K_IR_6.2 R301H/G/C/P loss-of-function mutations are located just C-terminal from the G-loop domain. These mutants display reduced surface expression when expressed in COSm6 or INS cells [51]. Interestingly, however, the R301A mutation displays normal expression at the plasma membrane [51]. At homologues positions, mutations have also been found in K_IR_1.1 [52] and K_IR_4.1 [53].

By use of homology comparison and structure guided mutagenesis, a common Golgi-export signal patch was found to be formed by a C-terminal stretch of hydrophobic residues and basic residues from the N-terminus [16,17]. The C-terminal stretch sequence is formed by residues SYxxxEIxW indicated in Figure 4. Two Bartter syndrome associated K_IR_1.1 (Y314C; L320P) and two Andersen–Tawil syndrome associated K_IR_2.1 (delSY; W322S) confirmed trafficking mutations have been described in this region [46,48,54]. Interestingly, the W residue is not conserved in the K_IR_7.1 channel protein, which may indicate K_IR_ subtype specific use of the entire Golgi-export signal motif. Two additional K_IR_1.1 mutations leading to altered trafficking, R324L and F325C have been located directly C-terminal from the Golgi-export signal stretch [48,52].

Fallen et al. [55] showed that mutations A198T and Y314C in the IgLD, located in the CTD of K_IR_1.1, are associated with defects in channel trafficking and gating, see also Section 6.1. Y314C is present within the C-terminal trafficking mutation hotspot and part of the Golgi-export signal as discussed above. If incorrectly folded, the aberrant K_IR_1.1 proteins will enter the endoplasmic-reticulum-associated protein degradation pathway [56].

### 5.2. Transmembrane Region 1 Mutation Hotspot

**Figure 5:**
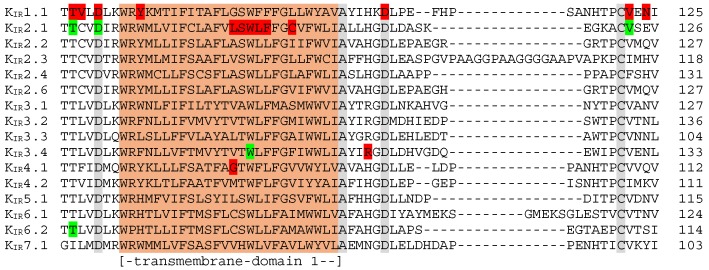
K_IR_1–7 sequence alignment of the transmembrane domain 1 mutation hotspot. Red: mutations associated with aberrant trafficking; Green: residues whose mutations are currently not related to impaired trafficking. Numbers at the right refer to the last amino-acid residue in the respective sequence shown. Conserved residues among all K_IR_ members are shaded gray. Location of the transmembrane domain 1 (orange) is indicated below the alignment.

Figure 5 depicts the alignment of transmembrane domain 1 and adjacent sequences, containing 15 trafficking associated mutations located in K_IR_1.1, K_IR_2.1, K_IR_3.4 or K_IR_4.1. Three mutations in the cytoplasmic domain, positioned just in front of the first transmembrane region in K_IR_1.1 (T71M, V72M and D74Y), have been demonstrated to strongly decrease plasma-membrane expression and mutant channels were retained in the cytoplasm [48,57]. However, membrane expression of T71M in *Xenopus* oocytes could be rescued by increasing the amount of injected RNA, in contrast to the other two mutations. For K_IR_1.1 T71M, mutations at the homologues positions were found in K_IR_2.1 (T75) [58,59,60,61,62] and K_IR_6.2 (T62) [63,64] associated with Andersen–Tawil syndrome and Familial hyperinsulinemic hypoglycemia type 2, respectively. The K_IR_2.1 T75R protein was observed at the plasma membrane upon overexpression in HL1 cells [60]. Moreover, T75A, T75R and T75M channel proteins were also expressed at the plasma membrane in *Xenopus* oocytes, HEK293 or COS-7 cells [58,61,65]. In contrast, impaired plasma-membrane localization of T75M K_IR_2.1 was observed in HEK293 cells in another study [62]. Two K_IR_2.1 mutations, i.e., D78G and D78Y, are at the equivalent position as D74 in K_IR_1.1, and also D78Y was found at the plasma membrane in *Xenopus* oocytes and HEK293 cells [59,65,66]. These comparisons indicate that findings on plasma-membrane expression may be influenced by the degree of overexpression and cell type. K_IR_2.1 T75 and D78 residues are positioned on the hydrophilic side of the slide helix that interacts with the cytoplasmic domain. The D78Y mutation disrupts this interaction [65].

Trafficking associated mutations in the highly conserved transmembrane region 1 are described for K_IR_1.1, K_IR_2.1 and K_IR_4.1 [46,48,67,68,69]. Expression of K_IR_1.1 Y79H in the *Xenopus* oocyte plasma-membrane increases upon increasing the amount of RNA injection by ten-fold [48]. The K_IR_2.1 L94P, Δ95-98 and K_IR_4.1 G77R channel proteins localize intracellularly [46,68,69]. The molecular mechanisms by which these mutations affect normal trafficking remain to be solved. However, interactions with wildtype subunits appear not to be affected and may explain the dominant negative properties of these mutations. The familial sinus node disease associated K_IR_3.4 W101C gain-of-function mutation is located at a position homologues to K_IR_2.1 W96 [70]. In an ectopic expression system, the K_IR_3.4 W101C protein is expressed at the plasma membrane, however it decreased surface expression of K_IR_3.1 when co-expressed [70].

Confirmed trafficking associated mutations C-terminal from the transmembrane region 1 are found in K_IR_1.1 and K_IR_3.4 [48,71,72]. K_IR_1.1 D108H and V122E mutants did not display membrane staining in *Xenopus* oocytes or HEK293 cells [48]. When comparing single channel characteristics with macroscopic currents, it was concluded that loss-of-function of K_IR_1.1 N124K was caused by a reduction of functional channels at the plasma membrane [71]. The K_IR_3.4 R115W mutation was obtained from aldosterone-producing adenoma linked to hyperaldosteronism, and displayed decreased plasma-membrane expression in HEK293 cells [72]. A mutation, at a position homologues to V122 in K_IR_1.1, has also been identified in K_IR_2.1 [59].

### 5.3. N-Terminal Golgi-Export Patch, K_IR_2.x ER Export, and K_IR_6.x ER Exit and Retention Signals

As indicated above, the so-called Golgi-export patch consists of interaction of a C-terminal and N-terminal domain [16,17]. Mutations in the C-terminal domain have been found (see Section 5.1). However, only few mutations have been described in the N-terminal part (K_IR_2.1 G52V; K_IR_2.6 R43C) that result in reduced plasma membrane expression by hampering Golgi export [73,74]. Thus far, no mutations of residue R20 in K_IR_2.3, which is required for Golgi export [17], or at the homologues position in any other K_IR_ channel protein have been identified.

K_IR_2.x channels share a short C-terminal ER-export signal (FCYENE) [10,11]. K_IR_3.2 contains N-terminal (DQDVESPV) and C-terminal (ELETEEEE) ER-export signals, whereas K_IR_3.4 possesses the N-terminal NQDMEIGV ER-export signal [12]. Remarkable, we did not encounter any trafficking associated mutations in any of these domains. In contrast, one mutation (E282K) was present in the di-acidic ER exit signal of K_IR_6.2 as discussed in Section 5.1. K_IR_6.x and SURx channel proteins contain a C-terminal ER-retention signal (RKR) [13]. Upon channel assembly, retention signals from both proteins are shielded, supporting subsequent ER-export. No trafficking associated mutations were found in these retention signals in K_IR_6.x channel proteins.

We therefore propose that mutations in Golgi-export domains have more severe clinical implications than mutations in ER-export/retention signals.

## 6. Structural Mapping of Trafficking Defect Causing Mutants

**Figure 6:**
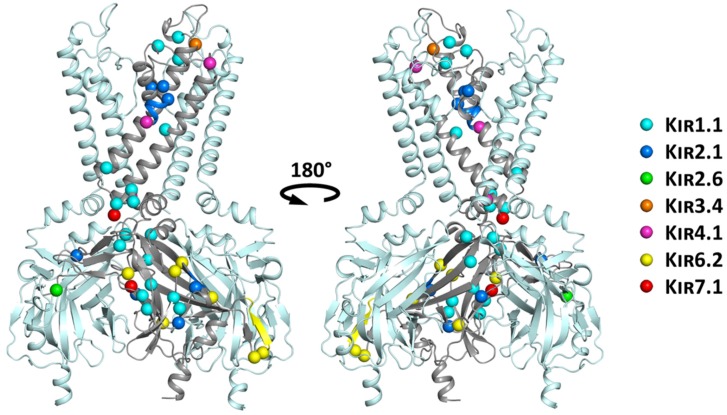
Structural mapping of trafficking mutants mapped on the K_IR_2.2 structure. For clarity reasons, only three of the four subunits are shown in side view, with the disease associated mutations highlighted in one subunit only. Mutations of different K_IR_ channel family members are color-coded and shown as spheres of their respective Cα atoms. Deletions are indicated by colored regions on the secondary structure elements.

Disease causing mutations associated with trafficking deficiencies were mapped onto the common structural scaffold of a recently published high resolution K_IR_2.2 structure [75]. As illustrated in Figure 6, mutations are globally distributed.

A group of mutations clusters at regions important for channel gating, including the PIP_2_ binding site (T71M, V72E, D74Y and Y79H in K_IR_1.1), the helix bundle crossing gate (A167V in K_IR_4.1; R162W/Q in K_IR_7.1) [76,77,78], as well as the G-loop gate (V302M, K_IR_2.1). It can be expected that these mutations have strong effects on the conformational equilibrium, thereby impairing normal protein function. It is likely that these mutants lead to structurally less-stable proteins, thereby making them more susceptible for degradation. Interestingly, 58% of the currently known trafficking defect causing mutations in K_IR_ channel proteins cluster in the cytoplasmic domain, which has been shown to be crucial for efficient folding in K_v_AP channels [79].

Another cluster of mutants (D108H, V122E and N124K in K_IR_1.1; R115W in K_IR_3.4; C140R in K_IR_4.1) is found on surface exposed loops of the channel. Except for C140R in K_IR_4.1 [68], which is part of a disulfide bridge [80], none of the mutations causes changes in polarity or is at important structural motifs, leaving it unclear why these mutants cause trafficking defects.

Mutations G77R in K_IR_4.1 [68] and C101R in K_IR_2.1, located on the membrane facing side and near the center of transmembrane helix M1, cause changes in the helical properties and hydrophobicity. It is thus conceivable that they severely affect helical stability and possibly membrane insertion. It has been shown in numerous studies that the cost for exposing arginine to lipid hydrocarbons is prohibitively high [81]. Interestingly, none of the identified disease mutations is located at the interface between subunits.

### 6.1. Structure-Based Hotspot in the IgLD Beta Barrel of the CTD

**Figure 7:**
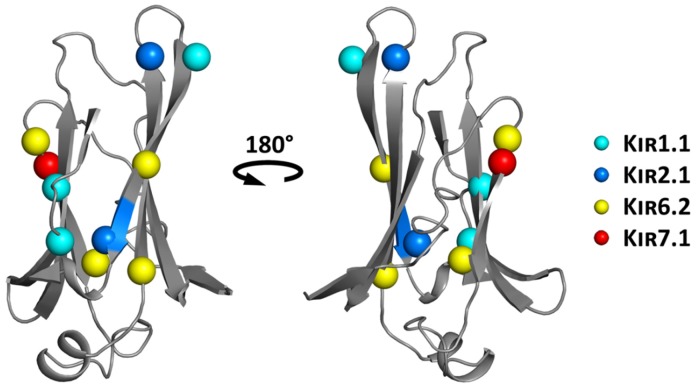
Structure-based IgLD hotspot (mapped on the K_IR_2.2 structure), with disease associated mutations highlighted. Mutations of the different family members are color-coded and shown as spheres of their respective Cα atoms.

Two antenatal Bartter syndrome loss-of-function mutations A198T and Y314C, located in the IgLD have been shown to impair forward trafficking and gating of K_IR_1.1 channels, possibly influencing the core stability of this domain [55]. Interestingly, a trafficking affecting mutation in a homologous position to K_IR_1.1 A198 has been identified in K_IR_2.6 (A200P) [74], and another mutation thus far not associated with trafficking has been identified in K_IR_6.2 (A187V). As shown in Figure 7, quite a large number of disease causing mutations, including A306T (implicated in trafficking) [48], R311W and L320P in K_IR_1.1 (no data on trafficking), S314-Y315 deletion in K_IR_2.1 (implicated in trafficking) [16], E282K (prevents ER-export and surface expression of the channel) [45] or L241P in K_IR_7.1 (implicated in trafficking) [78], have been reported in the literature. This, as well as previous work [55], suggests that this structural motif might be a crucial hotspot implicated in trafficking of K_IR_ channels.

## 7. Conclusions

Mutations in K_IR_ potassium ion channels associate with a variety of human diseases in which electrophysiological and potassium homeostasis aberrations are explaining etiology. Many of the mutations associate with abnormal, mostly decreased forward, ion channel trafficking. Trafficking associated mutations are present throughout the primary sequence, but they concentrate in cytoplasmic domains in which channel structures involved in Golgi-export are clinically more important than ER-export regions. Another group of mutations are found in regions important for gating and most likely affect protein folding and stability. Therefore, mutation associated K_IR_ trafficking defects are likely caused by 1) defective interaction with the trafficking machinery due to mutations in specific trafficking motifs, and 2) channel misfolding, destabilization and subsequent endoplasmic-reticulum-associated protein degradation due to mutations in residues important for channel structure.

## Figures and Tables

**Table 1:** Human diseases associated with abnormal K_IR_ channel function.

Protein	Gene	Syndrome/Disease Character (OMIM)^1^	Main Affected System(s)	Recent Review
K_IR_1.1	*KCNJ1*	Bartter syndrome, type 2 (241200)	Kidney; head; face; ear; eye; vascular; gastrointestinal; skeleton; skeletal muscle; CNS; platelets	[33]
K_IR_2.1	*KCNJ2*	Andersen syndrome (170390) Familial atrium fibrillation 9 (613980) Short QT syndrome 3 (609622)	Head; face; ear; eye; teeth; heart; skeleton; CNS	[34,35]
K_IR_2.2	*KCNJ12*	Non-described		
K_IR_2.3	*KCNJ4*	Non-described		
K_IR_2.4	*KCNJ14*	Non-described		
K_IR_2.6	*KCNJ18*	Thyrotoxic hypokalemic periodic paralysis (613239)	Cardiovascular; skeletal muscle; CNS; eye	[36]
K_IR_3.1	*KCNJ3*	Non-described		
K_IR_3.2	*KCNJ6*	Keppen–Lubinsky Syndrome (614098)	CNS; head; skin; skeleton; eye, face	No review available
K_IR_3.3	*KCNJ9*	Non-described		
K_IR_3.4	*KCNJ5*	Familial hyperaldosteronism 3 (613677) Long QT syndrome 13 (613485)	Cardiovascular; kidney; skeletal muscle	[39,40]
K_IR_4.1	*KCNJ10*	Digenic enlarged vestibular aqueduct (600791) EAST/SESAME syndrome (612780)	Ear (hearing); vascular; kidney; CNS	[41]
K_IR_4.2	*KCNJ15*	Non-described		
K_IR_5.1	*KCNJ16*	Non-described		
K_IR_6.1	*KCNJ8*	Cantú syndrome (239850)	Head; face; cardiovascular; skeleton; hair; CNS	[42]
K_IR_6.2	*KCNJ11*	Transient neonatal diabetes mellitus 3 (610582) Permanent neonatal diabetes with or without neurologic features (606176) Familial hyperinsulinemic hypoglycemia 2 (601820) Maturity-onset diabetes of the young 13 (616329) Susceptible to diabetes mellitus 2 (125853)	Pancreas (beta-cells); CNS	[43]
K_IR_7.1	*KCNJ13*	Leber congenital amaurosis 16 (614186) Snowflake vitreoretinal degeneration (193230)	Eye (retina)	[44]

OMIM^1^: OMIM^®^—Online Mendelian Inheritance in Man^®^ https://omim.org assessed on 24 July 2019, CNS, central neural system.

## Supplementary Materials

The following are available online at https://www.mdpi.com/2218-273X/9/11/650/s1, Figure S1: K_IR_1-7 sequence alignment.

## References

[1] Katz B. (1949). Les constantes électriques de la membrane du muscle. Arch. Sci. Physiol..

[2] Matsuda H., Saigusa A., Irisawa H. (1987). Ohmic conductance through the inwardly rectifying K channel and blocking by internal Mg^2+^. Nature.

[3] Lopatin A.N., Makhina E.N., Nichols C.G. (1994). Potassium channel block by cytoplasmic polyamines as the mechanism of intrinsic rectification. Nature.

[4] De Boer T.P., Houtman M.J., Compier M., Van der Heyden M.A. (2010). The mammalian K_IR_2. x inward rectifier ion channel family: Expression pattern and pathophysiology. Acta Physiol..

[5] Wang L., Chiamvimonvat N., Duff H.J. (1993). Interaction between selected sodium and potassium channel blockers in guinea pig papillary muscle. J. Pharmacol. Exp. Ther..

[6] Kokubun S., Nishimura M., Noma A., Irisawa H. (1982). Membrane currents in the rabbit atrioventricular node cell. Pflügers Arch..

[7] Yang J., Yu M., Jan Y.N., Jan L.Y. (1997). Stabilization of ion selectivity filter by pore loop ion pairs in an inwardly rectifying potassium channel. Proc. Natl. Acad. Sci. USA.

[8] Krapivinsky G., Gordon E.A., Wickman K., Velimirović B., Krapivinsky L., Clapham D.E. (1995). The G-protein-gated atrial K^+^ channel I_KACh_ is a heteromultimer of two inwardly rectifying K^+^-channel proteins. Nature.

[9] Lüscher C., Slesinger P.A. (2010). Emerging roles for G protein-gated inwardly rectifying potassium (GIRK) channels in health and disease. Nat. Rev. Neurosci..

[10] Ma D., Zerangue N., Lin Y.F., Collins A., Yu M., Jan Y.N., Jan L.Y. (2001). Role of ER export signals in controlling surface potassium channel numbers. Science.

[11] Stockklausner C., Ludwig J., Ruppersberg J.P., Klöcker N. (2001). A sequence motif responsible for ER export and surface expression of Kir2.0 inward rectifier K^+^ channels. FEBS Lett..

[12] Ma D., Zerangue N., Raab-Graham K., Fried S.R., Jan Y.N., Jan L.Y. (2002). Diverse trafficking patterns due to multiple traffic motifs in G protein-activated inwardly rectifying potassium channels from brain and heart. Neuron.

[13] Zerangue N., Schwappach B., Jan Y.N., Jan L.Y. (1999). A new ER trafficking signal regulates the subunit stoichiometry of plasma membrane K(ATP) channels. Neuron.

[14] Bundis F., Neagoe I., Schwappach B., Steinmeyer K. (2006). Involvement of Golgin-160 in cell surface transport of renal ROMK channel: Co-expression of Golgin-160 increases ROMK currents. Cell Physiol. Biochem..

[15] Taneja T.K., Ma D., Kim B.Y., Welling P.A. (2018). Golgin-97 Targets Ectopically Expressed Inward Rectifying Potassium Channel, Kir2.1, to the trans-Golgi Network in COS-7 Cells. Front. Physiol..

[16] Ma D., Taneja T.K., Hagen B.M., Kim B.Y., Ortega B., Lederer W.J., Welling P.A. (2011). Golgi export of the Kir2.1 channel is driven by a trafficking signal located within its tertiary structure. Cell.

[17] Li X., Ortega B., Kim B., Welling P.A. (2016). A Common Signal Patch Drives AP-1 Protein-dependent Golgi Export of Inwardly Rectifying Potassium Channels. J. Biol. Chem..

[18] Zeng W.Z., Babich V., Ortega B., Quigley R., White S.J., Welling P.A., Huang C.L. (2002). Evidence for endocytosis of ROMK potassium channel via clathrin-coated vesicles. Am. J. Physiol. Renal Physiol..

[19] Mackie T.D., Kim B.Y., Subramanya A.R., Bain D.J., O’Donnell A.F., Welling P.A., Brodsky J.L. (2018). The endosomal trafficking factors CORVET and ESCRT suppress plasma membrane residence of the renal outer medullary potassium channel (ROMK). J. Biol. Chem..

[20] Kolb A.R., Needham P.G., Rothenberg C., Guerriero C.J., Welling P.A., Brodsky JL. (2014). ESCRT regulates surface expression of the Kir2.1 potassium channel. Mol. Biol. Cell.

[21] Jansen J.A., de Boer T.P., Wolswinkel R., van Veen T.A., Vos M.A., van Rijen H.V.M., van der Heyden M.A.G. (2008). Lysosome mediated Kir2.1 breakdown directly influences inward rectifier current density. Biochem. Biophys. Res. Commun..

[22] Varkevisser R., Houtman M.J., Waasdorp M., Man J.C., Heukers R., Takanari H., Tieland R.G., van Bergen En Henegouwen P.M., Vos M.A., van der Heyden M.A. (2013). Inhibiting the clathrin-mediated endocytosis pathway rescues K(IR)2.1 downregulation by pentamidine. Pflugers Arch..

[23] Wang M.X., Su X.T., Wu P., Gao Z.X., Wang W.H., Staub O., Lin D.H. (2018). Kir5.1 regulates Nedd4-2-mediated ubiquitination of Kir4.1 in distal nephron. Am. J. Physiol. Renal Physiol..

[24] Leonoudakis D., Mailliard W., Wingerd K., Clegg D., Vandenberg C. (2001). Inward rectifier potassium channel Kir2.2 is associated with synapse-associated protein SAP97. J. Cell Sci..

[25] Leonoudakis D., Conti L.R., Radeke C.M., McGuire L.M., Vandenberg C.A. (2004). A multiprotein trafficking complex composed of SAP97, CASK, Veli, and Mint1 is associated with inward rectifier Kir2 potassium channels. J. Biol. Chem..

[26] Leonoudakis D., Conti L.R., Anderson S., Radeke C.M., McGuire L.M., Adams M.E., Froehner S.C., Yates J.R., Vandenberg C.A. (2004). Protein trafficking and anchoring complexes revealed by proteomic analysis of inward rectifier potassium channel (Kir2.x)-associated proteins. J. Biol. Chem..

[27] Pegan S., Tan J., Huang A., Slesinger P.A., Riek R., Choe S. (2007). NMR studies of interactions between C-terminal tail of Kir2.1 channel and PDZ1,2 domains of PSD95. Biochemistry.

[28] Brasko C., Hawkins V., De La Rocha I.C., Butt A.M. (2017). Expression of Kir4.1 and Kir5.1 inwardly rectifying potassium channels in oligodendrocytes, the myelinating cells of the CNS. Brain Struct. Funct..

[29] Tanemoto M., Fujita A., Higashi K., Kurachi Y. (2002). PSD-95 mediates formation of a functional homomeric Kir5.1 channel in the brain. Neuron.

[30] Horio Y., Hibino H., Inanobe A., Yamada M., Ishii M., Tada Y., Satoh E., Hata Y., Takai Y., Kurachi Y. (1997). Clustering and enhanced activity of an inwardly rectifying potassium channel, Kir4.1, by an anchoring protein, PSD-95/SAP90. J. Biol. Chem..

[31] Vaidyanathan R., Taffet S.M., Vikstrom K.L., Anumonwo J.M. (2010). Regulation of cardiac inward rectifier potassium current (I(K1)) by synapse-associated protein-97. J. Biol. Chem..

[32] Sampson L.J., Leyland M.L., Dart C. (2003). Direct interaction between the actin-binding protein filamin-A and the inwardly rectifying potassium channel, Kir2.1. J. Biol. Chem..

[33] Seyberth H.W., Weber S., Kömhoff M. (2017). Bartter’s and Gitelman’s syndrome. Curr. Opin. Pediatr..

[34] Nguyen H.L., Pieper G.H., Wilders R. (2013). Andersen-Tawil syndrome: Clinical and molecular aspects. Int. J. Cardiol..

[35] Hancox J.C., Whittaker D.G., Du C., Stuart A.G., Zhang H. (2018). Emerging therapeutic targets in the short QT syndrome. Expert Opin. Ther. Targets.

[36] Fialho D., Robert C.G., Emma M. (2018). Periodic paralysis. Handbook of Clinical Neurology.

[37] Masotti A., Uva P., Davis-Keppen L., Basel-Vanagaite L., Cohen L., Pisaneschi E., Celluzzi A., Bencivenga P., Fang M., Tian M. (2015). Keppen-Lubinsky syndrome is caused by mutations in the inwardly rectifying K+ channel encoded by KCNJ6. Am. J. Hum. Genet..

[38] Horvath G.A., Zhao Y., Tarailo-Graovac M., Boelman C., Gill H., Shyr C., Lee J., Blydt-Hansen I., Drögemöller B.I., Moreland J. (2018). Gain-of-function KCNJ6 Mutation in a Severe Hyperkinetic Movement Disorder Phenotype. Neuroscience.

[39] Korah H.E., Scholl U.I. (2015). An Update on Familial Hyperaldosteronism. Horm. Metab. Res..

[40] Bohnen M.S., Peng G., Robey S.H., Terrenoire C., Iyer V., Sampson K.J., Kass R.S. (2017). Molecular Pathophysiology of Congenital Long QT Syndrome. Physiol. Rev..

[41] Abdelhadi O., Iancu D., Stanescu H., Kleta R., Bockenhauer D. (2016). EAST syndrome: Clinical, pathophysiological, and genetic aspects of mutations in KCNJ10. Rare Dis..

[42] Nichols C.G., Singh G.K., Grange D.K. (2013). KATP channels and cardiovascular disease: Suddenly a syndrome. Circ. Res..

[43] Tinker A., Aziz Q., Li Y., Specterman M. (2018). ATP-Sensitive Potassium Channels and Their Physiological and Pathophysiological Roles. Compr. Physiol..

[44] Kumar M., Pattnaik B.R. (2014). Focus on Kir7.1: Physiology and channelopathy. Channels.

[45] Taneja T.K., Mankouri J., Karnik R., Kannan S., Smith A.J., Munsey T., Christesen H.B., Beech D.J., Sivaprasadarao A. (2009). Sar1-GTPase-dependent ER exit of KATP channels revealed by a mutation causing congenital hyperinsulinism. Hum. Mol. Genet..

[46] Bendahhou S., Donaldson M.R., Plaster N.M., Tristani-Firouzi M., Fu Y.H., Ptácek L.J. (2003). Defective potassium channel Kir2.1 trafficking underlies Andersen-Tawil syndrome. J. Biol. Chem..

[47] Ma D., Tang X.D., Rogers T.B., Welling P.A. (2007). An andersen-Tawil syndrome mutation in Kir2. 1 (V302M) alters the G-loop cytoplasmic K^+^ conduction pathway. J. Biol. Chem..

[48] Peters M., Ermert S., Jeck N., Derst C., Pechmann U., Weber S., Schlingmann K.P., Seyberth H.W., Waldegger S., Konrad M. (2003). Classification and rescue of ROMK mutations underlying hyperprostaglandin E syndrome/antenatal Bartter syndrome. Kidney Int..

[49] Choi B.O., Kim J., Suh B.C., Yu J.S., Sunwoo I.N., Kim S.J., Kim G.H., Chung K.W. (2007). Mutations of KCNJ2 gene associated with Andersen-Tawil syndrome in Korean families. J. Hum. Genet..

[50] Gloyn A.L., Pearson E.R., Antcliff J.F., Proks P., Bruining G.J., Slingerland A.S., Howard N., Srinivasan S., Silva J.M., Molnes J. (2004). Activating mutations in the gene encoding the ATP-sensitive potassium-channel subunit Kir6.2 and permanent neonatal diabetes. N. Engl. J. Med..

[51] Lin Y.W., Bushman J.D., Yan F.F., Haidar S., MacMullen C., Ganguly A., Stanley C.A., Shyng S.L. (2008). Destabilization of ATP-sensitive potassium channel activity by novel KCNJ11 mutations identified in congenital hyperinsulinism. J. Biol. Chem..

[52] Schulte U., Hahn H., Konrad M., Jeck N., Derst C., Wild K., Weidemann S., Ruppersberg J.P., Fakler B., Ludwig J. (1999). pH gating of ROMK (K(ir)1.1) channels: Control by an Arg-Lys-Arg triad disrupted in antenatal Bartter syndrome. Proc. Natl. Acad. Sci. USA.

[53] Scholl U.I., Choi M., Liu T., Ramaekers V.T., Häusler M.G., Grimmer J., Tobe S.W., Farhi A., Nelson-Williams C., Lifton R.P. (2009). Seizures, sensorineural deafness, ataxia, mental retardation, and electrolyte imbalance (SeSAME syndrome) caused by mutations in KCNJ10. Proc. Natl. Acad. Sci. USA..

[54] Limberg M.M., Zumhagen S., Netter M.F., Coffey A.J., Grace A., Rogers J., Böckelmann D., Rinné S., Stallmeyer B., Decher N. (2013). Non dominant-negative KCNJ2 gene mutations leading to Andersen-Tawil syndrome with an isolated cardiac phenotype. Basic Res. Cardiol..

[55] Fallen K., Banerjee S., Sheehan J., Addison D., Lewis L.M., Meiler J., Denton J.S. (2009). The Kir channel immunoglobulin domain is essential for Kir1.1 (ROMK) thermodynamic stability, trafficking and gating. Channels.

[56] O’Donnell B.M., Mackie T.D., Subramanya A.R., Brodsky J.L. (2017). Endoplasmic reticulum-associated degradation of the renal potassium channel, ROMK, leads to type II Bartter syndrome. J. Biol. Chem..

[57] Károlyi L., Konrad M., Köckerling A., Ziegler A., Zimmermann D.K., Roth B., Wieg C., Grzeschik K.H., Koch M.C., Seyberth H.W. (1997). Mutations in the gene encoding the inwardly-rectifying renal potassium channel, ROMK, cause the antenatal variant of Bartter syndrome: Evidence for genetic heterogeneity. International Collaborative Study Group for Bartter-like Syndromes. Hum. Mol. Genet..

[58] Fodstad H., Swan H., Auberson M., Gautschi I., Loffing J., Schild L., Kontula K. (2004). Loss-of-function mutations of the K^+^ channel gene KCNJ2 constitute a rare cause of long QT syndrome. J. Mol. Cell. Cardiol..

[59] Davies N.P., Imbrici P., Fialho D., Herd C., Bilsland L.G., Weber A., Mueller R., Hilton-Jones D., Ealing J., Boothman B.R. (2005). Andersen-Tawil syndrome: New potassium channel mutations and possible phenotypic variation. Neurology.

[60] Lu C.W., Lin J.H., Rajawat Y.S., Jerng H., Rami T.G., Sanchez X., DeFreitas G., Carabello B., DeMayo F., Kearney D.L. (2006). Functional and clinical characterization of a mutation in KCNJ2 associated with Andersen-Tawil syndrome. J. Med. Genet..

[61] Eckhardt L.L., Farley A.L., Rodriguez E., Ruwaldt K., Hammill D., Tester D.J., Ackerman M.J., Makielski J.C. (2007). KCNJ2 mutations in arrhythmia patients referred for LQT testing: A mutation T305A with novel effect on rectification properties. Heart Rhythm..

[62] Tani Y., Miura D., Kurokawa J., Nakamura K., Ouchida M., Shimizu K., Ohe T., Furukawa T. (2007). T75M-KCNJ2 mutation causing Andersen-Tawil syndrome enhances inward rectification by changing Mg^2+^ sensitivity. J. Mol. Cell. Cardiol..

[63] Snider K.E., Becker S., Boyajian L., Shyng S.L., MacMullen C., Hughes N., Ganapathy K., Bhatti T., Stanley C.A., Ganguly A. (2013). Genotype and phenotype correlations in 417 children with congenital hyperinsulinism. J. Clin. Endocrinol. Metab..

[64] Mohnike K., Wieland I., Barthlen W., Vogelgesang S., Empting S., Mohnike W., Meissner T., Zenker M. (2014). Clinical and genetic evaluation of patients with KATP channel mutations from the German registry for congenital hyperinsulinism. Horm. Res. Paediatr..

[65] Decher N., Renigunta V., Zuzarte M., Soom M., Heinemann S.H., Timothy K.W., Keating M.T., Daut J., Sanguinetti M.C., Splawski I. (2007). Impaired interaction between the slide helix and the C-terminus of Kir2.1: A novel mechanism of Andersen syndrome. Cardiovasc. Res..

[66] Yoon G., Oberoi S., Tristani-Firouzi M., Etheridge S.P., Quitania L., Kramer J.H., Miller B.L., Fu Y.H., Ptácek L.J. (2006). Andersen-Tawil syndrome: Prospective cohort analysis and expansion of the phenotype. Am. J. Med. Genet. A.

[67] Ballester L.Y., Benson D.W., Wong B., Law I.H., Mathews K.D., Vanoye C.G., George A.L. Jr. (2006). Trafficking-competent and trafficking-defective KCNJ2 mutations in Andersen syndrome. Hum. Mutat..

[68] Williams D.M., Lopes C.M., Rosenhouse-Dantsker A., Connelly H.L., Matavel A., O-Uchi J., McBeath E., Gray D.A. (2010). Molecular basis of decreased Kir4.1 function in SeSAME/EAST syndrome. J. Am. Soc. Nephrol..

[69] Takeda I., Takahashi T., Ueno H., Morino H., Ochi K., Nakamura T., Hosomi N., Kawakami H., Hashimoto K., Matsumoto M. (2013). Autosomal recessive Andersen–Tawil syndrome with a novel mutation L94P in Kir2.1. Neurol. Clin. Neurosci..

[70] Kuß J., Stallmeyer B., Goldstein M., Rinné S., Pees C., Zumhagen S., Seebohm G., Decher N., Pott L., Kienitz M.C. (2019). Familial Sinus Node Disease Caused by a Gain of GIRK (G-Protein Activated Inwardly Rectifying K+ Channel) Channel Function. Circ. Genom. Precis. Med..

[71] Derst C., Wischmeyer E., Preisig-Müller R., Spauschus A., Konrad M., Hensen P., Jeck N., Seyberth H.W., Daut J., Karschin A. (1998). A hyperprostaglandin E syndrome mutation in *Kir1. 1* (renal outer medullary potassium) channels reveals a crucial residue for channel function in Kir1. 3 channels. J. Biol. Chem..

[72] Cheng C.J., Sung C.C., Wu S.T., Lin Y.C., Sytwu H.K., Huang C.L., Lin S.H. (2015). Novel KCNJ5 mutations in sporadic aldosterone-producing adenoma reduce Kir3.4 membrane abundance. J. Clin. Endocrinol. Metab..

[73] Gélinas R., El Khoury N., Chaix M.A., Beauchamp C., Alikashani A., Ethier N., Boucher G., Villeneuve L., Robb L., Latour F. (2017). Characterization of a Human Induced Pluripotent Stem Cell-Derived Cardiomyocyte Model for the Study of Variant Pathogenicity: Validation of a KCNJ2 Mutation. Circ. Cardiovasc. Genet..

[74] Cheng C.J., Lin S.H., Lo Y.F., Yang S.S., Hsu Y.J., Cannon S.C., Huang C.L. (2011). Identification and functional characterization of Kir2.6 mutations associated with non-familial hypokalemic periodic paralysis. J. Biol. Chem..

[75] Lee S.J., Ren F., Zangerl-Plessl E.M., Heyman S., Stary-Weinzinger A., Yuan P., Nichols C.G. (2016). Structural basis of control of inward rectifier Kir2 channel gating by bulk anionic phospholipids. J. Gen. Physiol..

[76] Tanemoto M., Abe T., Uchida S., Kawahara K. (2014). Mislocalization of K^+^ channels causes the renal salt wasting in EAST/SeSAME syndrome. FEBS Lett..

[77] Pattnaik B.R., Tokarz S., Asuma M.P., Schroeder T., Sharma A., Mitchell J.C., Edwards A.O., Pillers D.A. (2013). Snowflake vitreoretinal degeneration (SVD) mutation R162W provides new insights into Kir7.1 ion channel structure and function. PLoS ONE.

[78] Sergouniotis P.I., Davidson A.E., Mackay D.S., Li Z., Yang X., Plagnol V., Moore A.T., Webster A.R. (2011). Recessive mutations in KCNJ13, encoding an inwardly rectifying potassium channel subunit, cause leber congenital amaurosis. Am. J. Hum. Genet..

[79] McDonald S.K., Levitz T.S., Valiyaveetil F.I. (2019). A Shared Mechanism for the Folding of Voltage-Gated K+ Channels. Biochemistry.

[80] Cho H.C., Tsushima R.G., Nguyen T.T., Guy H.R., Backx P.H. (2000). Two critical cysteine residues implicated in disulfide bond formation and proper folding of Kir2. 1. Biochemistry.

[81] Hristova K., Wimley W.C. (2011). A look at arginine in membranes. J. Membr. Biol..

