# Supplementary material for: Disease Associated Mutations in KIR Proteins Linked to Aberrant Inward Rectifier Channel Trafficking
Source: Biomolecules. 2019 Oct 25;9(11):650. doi: 10.3390/biom9110650 (PMC6920955; doi:10.3390/biom9110650)
Supplement: Supplementary file 1 [file biomolecules-09-00650-s001.pdf]

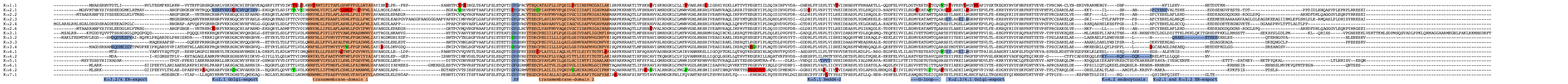

Residues which mutation is associated with trafficking defects
Mutations in residues with non-described, or conflicting, effect on trafficking, homology might suggest trafficking defect of the channel in response to the mutation

Figure S1
